# Phenotypic Expression of Diaspores Indicates Genetic Diversity in Natural Populations of *Spondias tuberosa* (Anacardiaceae)

**DOI:** 10.3390/biology14121641

**Published:** 2025-11-21

**Authors:** João Henrique Constantino Sales Silva, Joyce Naiara da Silva, Caroline Marques Rodrigues, Eduardo Luã Fernandes da Silva, Luís Gustavo Alves de Almeida, Maria Karoline Ferreira Bernardo, Kaline Lígia do Nascimento, Ruth da Silva Ramos, Naysa Flávia Ferreira do Nascimento, Edna Ursulino Alves

**Affiliations:** Department of Plant Science and Environmental Sciences, Center for Agrarian Sciences, Federal University of Paraíba, University Campus II, Areia 58397-000, PB, Brazil; joicenaiara@hotmail.com (J.N.d.S.); marxcarol48@hotmail.com (C.M.R.); eduardo.eng.fernandes@gmail.com (E.L.F.d.S.); luis.alves2@academico.ufpb.br (L.G.A.d.A.); karolinebernardo249@gmail.com (M.K.F.B.); kldn@academico.ufpb.br (K.L.d.N.); ruthsr01@gmail.com (R.d.S.R.); naysa.flavia@academico.ufpb.br (N.F.F.d.N.); ursulinoalves@hotmail.com (E.U.A.)

**Keywords:** bioeconomy, Caatinga, conservation, morphological diversity, umbuzeiro

## Abstract

The aim of this study was to assess phenotypic variation and its implications for genetic diversity in natural populations of *Spondias tuberosa*. Fruits were harvested from 38 mother plants from three natural populations, and the physical traits of the fruits, endocarps, and physiological quality of the seeds were evaluated in a completely randomized design with four replicates of 25 fruits per genotype. The data were subjected to analysis of variance, and the means were grouped via the Scott–Knott method. Genetic diversity was evaluated via UPGMA and Tocher clustering methods, which are based on the Mahalanobis distance (D^2^) and canonical variables, whereas the relative importance of the characters was evaluated via the Singh method. The results indicated high phenotypic variability, with fruit length was the main discriminant trait among genotypes. The groupings formed six (UPGMA) and 12 groups (Tocher), indicating greater divergence within populations. The analysis of phenotypic data provides a solid basis for understanding the ecological dynamics of and supporting sustainable management practices for *S. tuberosa*, in line with bioeconomic objectives. This study highlights the importance of preserving phenotypic variability in populations, which is essential for the adaptation of species to environmental changes and for improving local bioeconomies.

## Simple Summary

The umbuzeiro (*Spondias tuberosa*) is a very important tree in the Brazilian semi-arid region, and the genetic differences that exist between its populations are a valuable resource for ensuring both the conservation of the species and the improvement of fruit production. In this study, fruits from 38 trees from three natural populations were analyzed, observing the size, weight, and quality of the seeds. The research showed that there is great diversity among umbuzeiro trees, especially in fruit length, which was the most striking characteristic in differentiating the trees. Several distinct groups of plants were identified, indicating that there is great variety within each population. This diversity is essential, as it increases the species’ chances of adapting to climate change and contributes to more sustainable cultivation practices, in addition to strengthening the local economy based on umbu.

## 1. Introduction

Seasonally dry tropical forests (SDTFs) are ecosystems of great global importance, but they still receive less scientific attention than tropical rainforests, despite facing similar threats [1]. In Brazil, they are mainly located in the Northeast, within the Caatinga biome, one of the largest and most biodiverse dry tropical regions in the world [2]. Characterized by steppe savanna vegetation, with deciduous trees and shrubs adapted to drought, the Caatinga is home to high levels of endemism and adaptations resulting from long evolutionary processes [3]. However, the genetic diversity of wild plants has been declining due to habitat loss, climate change, fragmentation, overgrazing, and inappropriate management practices [4,5], making it essential to assess genetic quality, especially of seeds, for conservation and propagation [6,7,8].

Genetic studies show that species variability and appropriate management practices increase the resilience and sustainability of local populations [9,10,11]. Analysis of the morphological and physiological traits of fruits and seeds allows the identification of promising genotypes and understanding of processes such as germination and dormancy, which are fundamental to ecological restoration [12]. The genetic diversity observed by morphological descriptors reflects adaptation to local environmental conditions and guides the selection of more adapted and genetically diverse genotypes, promoting restored ecosystems that are more resilient and sustainable in the long term [13,14].

The genus *Spondias* L. belongs to the Anacardiaceae family and comprises 18 species, ten of which are Neotropical, occurring from Mexico to southeastern Brazil; one species is in Madagascar, and the rest is native to tropical Asia and the South Pacific [15]. The umbuzeiro (*Spondias tuberosa* Arruda) is a native and endemic plant to Brazil, especially the Caatinga Biome, and plays a crucial role in sustainable rural development in the semiarid region of Brazil because of its economic, social, and ecological importance [16]. Its fruits are appreciated for their exotic sweet–sour taste and nutritional value and are rich in vitamin C, carotenoids, and minerals, which are sold fresh and processed, reaching national and international markets [17,18].

Despite recent advances, there are still significant gaps in knowledge about the genetic variability of *S. tuberosa* in natural populations, especially regarding the relationship between phenotypic traits of diaspores and underlying genetic diversity. The morphological and physiological traits of fruits and seeds are indirect but effective indicators of genetic variability, as they reflect the expression of genes related to environmental adaptation and reproductive success of plants [12]. Previous molecular studies using ISSR (Inter-simple Sequence Repeat) [10,11], RAPD (Random Amplified Polymorphic DNA) [19], AFLP (Amplified Fragment Length Polymorphism) [20], and SNP (Single-Nucleotide Polymorphisms) [21] markers have already demonstrated significant intra– and interpopulation variability, reinforcing the need to integrate morphological and physiological approaches to fully understand the genetic structure and conservation potential of the species.

Given the importance of *Spondias tuberosa* in semiarid region and its adaptive capacity, the objective of this study was to analyze genetic variability based on the phenotypic and physiological traits of diaspores, aimed at supporting management strategies for the conservation and enhancement of natural populations.

## 2. Materials and Methods

### 2.1. Fruit Collection Site

**Figure 1:**
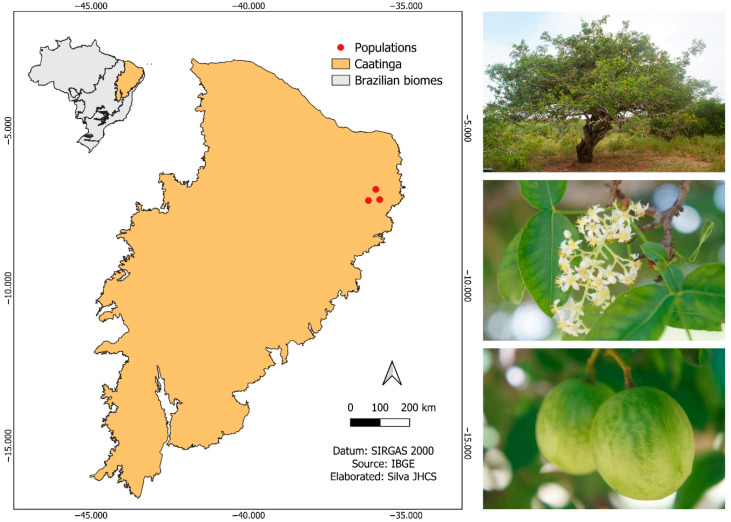
Locations of *Spondias tuberosa* Arruda (Anacardiaceae) fruit collection areas in the Caatinga, Brazil.

The fruits of *S. tuberosa* were collected from 38 naturally occurring parent plants of the species in the municipalities of Algodão de Jandaíra, Boa Vista, and São José da Mata (district of the municipality of Campina Grande) in the state of Paraíba, Northeast Brazil. The three populations of *S. tuberosa* are located in the Caatinga phytogeographic domain (Figure 1). The region’s climate is hot semiarid (BSh), according to the Köppen–Geiger classification, with high temperatures and scarce and irregular rainfall. The region’s phytophysionomy is characterized by xerophytic vegetation, composed of shrubs, cacti, low trees, and grasses, adapted to the semiarid climate and low-fertility soils. The number of populations and mother plants were defined considering the availability of reproductive individuals in the selected areas, covering different soil and climate conditions and degrees of environmental disturbance, in order to capture the genetic variability existing in the region.

The environmental characteristics of the three collection areas are described in Table 1, emphasizing that there are similarities between populations in terms of landscape management areas, with individuals present in both managed and unmanaged areas in the composition of the populations.

In this study, each parent tree was georeferenced with a GPS device to record its exact coordinates. In addition, all the sampled individuals were spaced at least 20 m apart to reduce sampling bias, taking into account their allogamous mode of reproduction [22,23].

The collections were carried out between March and May 2023/2024, during the period when the fruits were ripe, varying according to the plant population. For selection, fruits with a mixture of green, yellow, and/or orange tones were considered ripe [24]. The fruits were collected randomly from both the ground and the canopy of each parent plant. They were then stored in plastic bags and taken to the Seed Analysis Laboratory (LAS) of the Department of Plant Science at the Center for Agricultural Sciences of the Federal University of Paraíba (DFCA–CCA/UFPB) for biometric and physiological analysis.

### 2.2. Biometrics of Fruits and Endocarps

For the biometric characterization of fruits and endocarps, 100 ripe fruits from each parent plant were used, and the length (mm), diameter (mm), and fresh weight (g) of the fruits were determined. After the measurements, the fruits were pulped with a serrated knife, and the endocarps were washed in running water to remove excess pulp. A coarse mesh sieve was used to facilitate maceration. The endocarps were then allowed to dry on plastic trays on a greenhouse bench for 72 h at room temperature, after which their length (mm), width (mm), thickness (mm), and mass (g) were evaluated. The dimensions were measured with a digital caliper with an accuracy of 0.01 mm, whereas the fresh weight was determined with an analytical balance with an accuracy of 0.001 g.

### 2.3. Seedling Emergence Test

Although dormancy in *S. tuberosa* seeds is due to mechanical restrictions, no pre-germination or phytosanitary treatment has been applied. Sowing was performed with four replicates of 25 endocarps distributed in two plastic trays measuring 60 × 37 × 14 cm (length × width × height) and filled with sterilized medium-grain sand, leaving a 3.0 cm border. The test was set up in a greenhouse, with sowing performed at a depth of 3.0 cm. The temperature and humidity were monitored daily with a thermohygrometer, which recorded an average temperature of 32 °C and a relative humidity of 60%. Substrate moisture was maintained by manual watering, which was performed daily as needed.

To consider seedling emergence, the criterion of counting those with cotyledons completely above the substrate was adopted, with the results expressed as a percentage. In addition to the emergence test, the emergence speed index (ESI) and mean emergence time (MET) were also determined through daily counts until the 90th day after sowing and were calculated according to the equations proposed by Maguire [25] and Edmond and Drapalla [26], respectively.

### 2.4. Design and Statistical Analysis

The experimental design adopted was completely randomized, with four replicates of 25 fruits for each genotype. The statistical model used is described by the following equation: $Y_{ij}=µ+G_{i}+e_{ij}$, where i is the genotype indicator; j is the repetition indicator; $Y_{ij}$ represents the observed value in the genotype characteristic; µ is the overall mean; $G_{i}$ is the genotype effect; $e_{ij}$ is the random error.

The quantitative data were subjected to tests of normality and homoscedasticity of residual variances, followed by analysis of variance (ANOVA), with subsequent grouping of means by the Scott–Knott test (*p* < 0.05). The variables analyzed among the three plant populations are represented by boxplots.

For genetic divergence analysis, we used the Tocher clustering method, which is based on the generalized Mahalanobis distance (D^2^) [27], and the unweighted pair group method with arithmetic mean (UPGMA) hierarchical method. The determination of the cutoff point of the generated dendrogram, as well as the definition of the number of groups, were estimated according to Mojena’s method [28] on the basis of the relative size of the distances in the dendrogram. The relative importance of the traits was determined via Singh’s method [29], and then a Pearson correlation (*r*) was performed to evaluate associations between the variables.

Genetic parameters and their estimators were analyzed for each trait via the following mathematical expressions [30]:

(a) Phenotypic variance: ${\hat{\sigma }}_{f}^{2}=\frac{{QM}_{g}}{k}$
(b) Environmental variance: ${\hat{\sigma }}_{e}^{2}=\frac{{QM}_{r}}{k}$
(c) Genetic variance: ${\hat{\sigma }}_{g}^{2}=\frac{{QM}_{g}-{QM}_{r}}{k}$

where *QM_g_* and *QM_r_* correspond to the mean squares of the genotype and error, respectively, and *k* is the number of replicates. From these components, the following genetic parameters were estimated:

(d) Heritability in the broad sense: $h^{2}=\frac{{\hat{\sigma }}_{g}^{2}}{{\hat{\sigma }}_{f}^{2}}$
(e) Coefficient of genetic variation: ${CV}_{g}=\frac{\sqrt{\sigma_{g}}}{m}\times 100$
(f) Coefficient of environmental variation: ${CV}_{e}=\frac{\sqrt{\sigma_{r}}}{m}\times 100$
(g) Ratio $\frac{{CV}_{g}}{{CV}_{e}}$
(h) Pearson correlation (*r*): $r=\frac{cov(x,y\}}{s_{x}s_{y}}$

where r: Pearson correlation coefficient; cov(x,y}: covariance between variables *x* and y; $s_{x}$: standard deviation of variable *x*; $s_{y}$ standard deviation of variable y.

Statistical analyses were performed via Genes software (version 1990.2023.15) [31] and R (version 4.2.1) [32] via the ScottKnott [33], candisc [34], biotools [35] and factoextra [36] packages.

## 3. Results

The treatment effects were significant according to the *F* test (*p* < 0.01) for all the traits (Table 2), indicating the existence of genetic variability among the genotypes evaluated. The heredity values were high, above 90% for all the variables, except for the mean emergence time (MET), whose h^2^ value was 63.10%. The ratio between the genetic and environmental coefficients of variation (CVg/CVe) was greater than 1 for all the traits except for MET, indicating a favorable situation for selection. The coefficients of variation (CVs) of the experiment ranged from 2.17 to 48.75%. The highest values were recorded for traits related to seed physiological quality, whose values ranged from 36.41 to 48.75%. This significant variation is due to the wide range of data for these traits; in contrast, the CV values for the other traits were less than 7%.

The averages of the phenotypic traits of the genotypes can be found in Table S1 (Supplementary Materials). Considering the results obtained via the Scott-Knott test (*p* < 0.05), the genotypes were grouped into up to nine classes, varying according to the traits analyzed.

**Figure 2:**
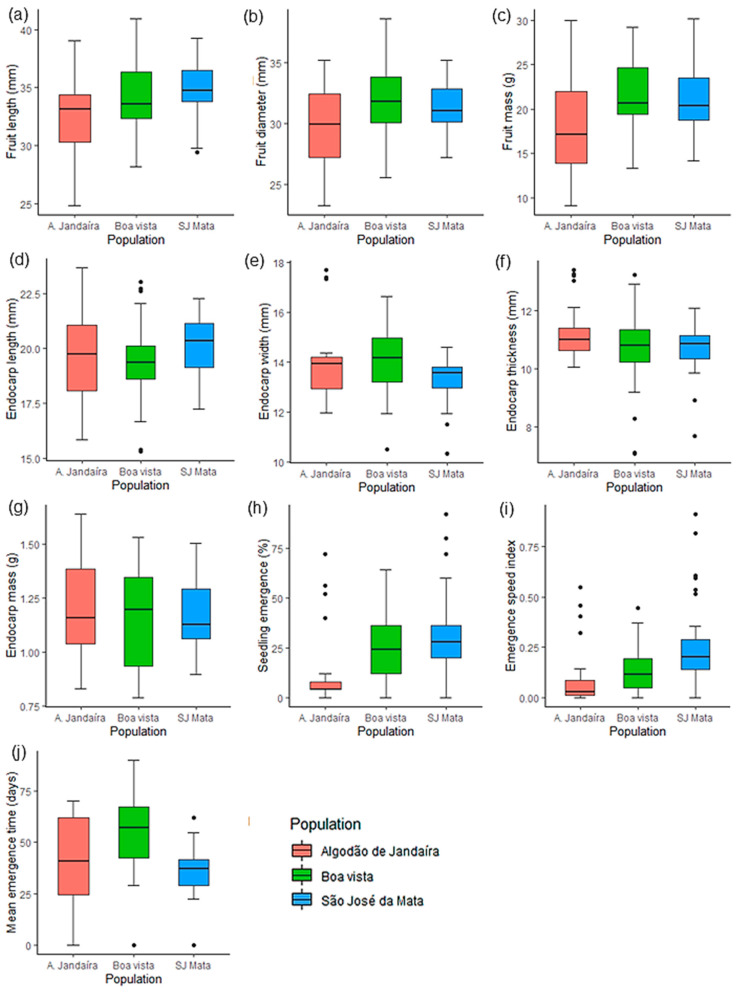
Boxplot analysis of the morphophysiological traits of *Spondias tuberosa* Arruda (Anacardiaceae) diaspores from three natural populations in Caatinga areas in Paraíba, Brazil. (**a**): fruit length (mm); (**b**): fruit diameter (mm); (**c**): fresh fruit mass (g); (**d**): endocarp length (mm); (**e**): endocarp width (mm); (**f**): endocarp thickness (mm); (**g**): endocarp mass (g); (**h**): seedling emergence (%); (**i**): emergence speed index (ESI); (**j**): mean emergence time (MET—days). The narrowest portion within each box represents the median; box notch boundaries indicate the median confidence interval; lower and upper bounds of the box show the lower and upper quartiles, respectively; vertical bars represent the maximum and minimum values; isolated points outside these limits are considered outliers.

When the phenotypic traits of the three *S. tuberosa* populations were analyzed via boxplots (Figure 2), a general pattern was observed that indicates lower genetic variability among the parent plants of the São José da Mata population, as evidenced by the reduced range of data from the collected diaspores. The length of the fruits was similar across the three plant populations, with medians ranging from 33.1 to 34.7 mm (Figure 2a). However, in the Boa Vista and São José da Mata plant populations, the medians were greater for fruit diameter and mass, with values of 31.8 mm and 31 mm in diameter and 20.6 g and 20.3 g in mass, respectively; in contrast, the values for the Algodão de Jandaíra plant population were lower, with a diameter of 29.9 mm and a mass of 17.2 g (Figure 2b,c). Notably, the largest fruits were observed in the Boa Vista plant population, with maximum dimensions of 40.9 × 38.6 mm (length × diameter) (Figure 2a,b).

With respect to the physical traits of the endocarps, small variations were found between populations: length varied from 19.3 to 20.3 mm; width ranged from 13.6 to 14.2 mm; thickness ranged from 10.8 to 11 mm; and endocarp mass ranged from approximately 1.12 to 1.20 g (Figure 2d–g). The greatest differences were observed in traits related to seed physiological quality, with seedling emergence rates significantly higher when originating from diaspores from the Boa Vista (25%) and São José da Mata (30%) plant populations, compared with 11% emergence of seedlings from diaspores of the Algodão de Jandaíra population (Figure 2h). This trend was also reflected in the emergence speed index (ESI), whose averages for these populations were 35.71% and 62.5% higher than those of the Algodão de Jandaíra plant population (Figure 2i). With respect to the mean emergence time (MET), the seedlings from the populations emerged at different intervals: approximately 36 days for São José da Mata, 40 days for Algodão de Jandaíra, and 54 days for Boa Vista (Figure 2j).

The Tocher optimization method, which is based on the Mahalanobis distance, allows the genotypes studied to be separated into 12 groups (Table 3), demonstrating that there is high variability among *S. tuberosa* parent plants in terms of the traits evaluated. Groups I and IV were formed by genotypes from the three plant populations, whereas group II was formed by genotypes from the São José da Mata and Boa Vista plant populations. Group III included genotypes from the Boa Vista plant population, and group V included genotypes from the Boa Vista and Algodão de Jandaíra plant populations. Finally, groups VI, VII, VIII, IX, X, XI, and XII are each composed of a single genotype, and it is important to note that groups I and II together account for approximately 55% of the genotypes evaluated.

**Figure 3:**
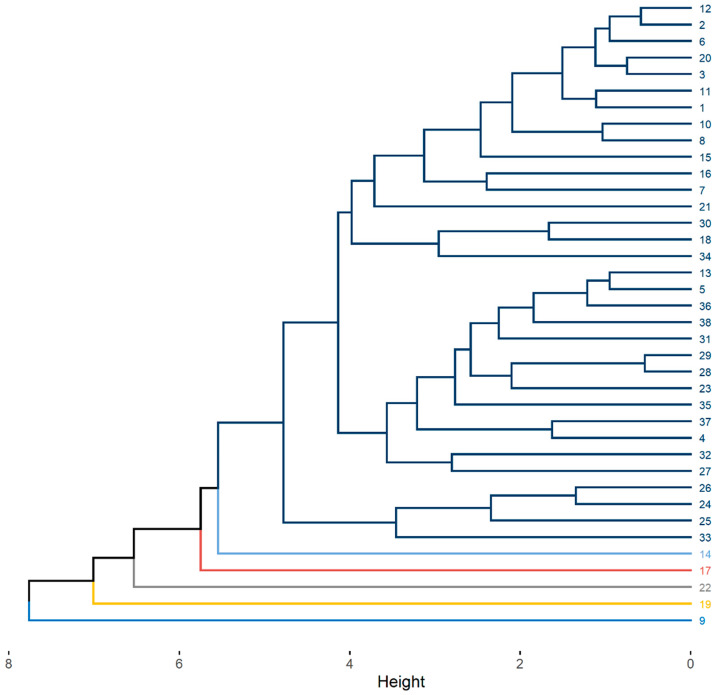
Dendrogram generated via UPGMA (standardized Euclidean distance) based on 10 phenotypic and physiological traits of *Spondias tuberosa* diaspores. Cutoff point = 5.1 (Mojena’s method) groups genotypes into 6 clusters (distinct colors). Genotype numbering: 1–13 = São José da Mata (SJ Mata), 14–21 = Algodão de Jandaíra (AJ), 22–38 = Boa Vista (BV). Single-genotype clusters (divergent genotypes) are labeled in bold: 9 (SJ Mata), 14 (AJ), 17 (AJ), 19 (AJ), and 22 (BV).

The dendrogram generated by the unweighted pair group method with arithmetic mean (UPGMA) clustering method, which uses standardized Euclidean distance, is shown in Figure 3. In accordance with Mojena’s method [28], the dendrogram cutoff point was set at 5.1, resulting in the formation of six groups, each represented by a different color. Of these, five groups consisted of only one individual: genotypes 14, 17, and 19 from the Jandaíra cotton plant population; genotype 22 from the Boa Vista plant population; and genotype 9 from the São José da Mata population. The remaining parent plants were grouped into a single group, which comprised 86.8% of the individuals, including genotypes collected from the three populations.

Among the Tocher and UPGMA methods, a difference was observed in the number of groups formed, with the former generating 12 groups and the latter resulting in only 6. However, it is important to note that genotypes 9, 14, 17, and 19 were grouped separately in both methods, forming a single group each. This finding indicates a similarity between the two methods in the formation of groups of the most divergent genotypes (Table 3; Figure 3). Detailed information on the average, minimum, and maximum values for each characteristic, for the groups formed by the Tocher and UPGMA methods, can be found in Table A1 and Table A2, respectively.

**Figure 4:**
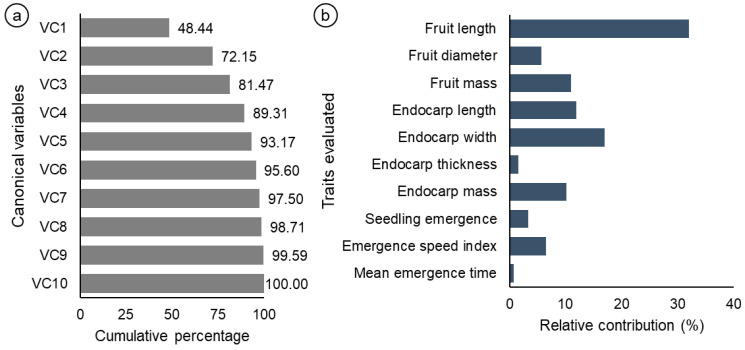
Analyses of the weighting coefficients obtained from the canonical variables (CV) of the eigenvalue estimates (**a**). Estimates of the relative contribution of each variable to genetic divergence on the basis of the calculation of Mahalanobis distances, according to Singh’s criterion (**b**).

Analysis of the canonical variables revealed that the first three accumulated variables explained more than 80% of the total variation (Figure 4a). Figure 4b shows the relative importance of the 10 traits evaluated, according to Singh’s method [29]. Among the variables analyzed, fruit length accounted for 32% of the total dissimilarity, followed by endocarp width, with 17% (Figure 4b). On the other hand, the mean emergence time was the characteristic with the lowest relative contribution to genetic divergence, at only 0.72%. These results indicate that specific traits are more efficient in explaining the dissimilarity observed between the evaluated genotypes.

The values obtained via Pearson’s correlation (Table 4) indicated significant and positive associations between the dimensions and masses of the fruits and endocarps, with the exception of the relationships between fruit length, endocarp width, and thickness. In addition, there were positive and significant correlations between seedling emergence and the emergence speed index (ESI) and fruit length, as well as between endocarp length and mass. On the other hand, there were no significant associations between the mean emergence time (MET) and the traits evaluated, except for ESI, which was significantly negatively correlated. These correlations were expected since larger fruits tend to be heavier. Similarly, heavier seeds generally store greater amounts of reserves, resulting in better germination and vigor.

## 4. Discussion

This study investigated the phenotypic variability between and within natural populations of *Spondias tuberosa*, and the results revealed high genetic diversity among the individuals analyzed, suggesting that the selection of the most promising individuals and the traits that contributed the most to this dissimilarity. This finding adds to the growing body of data on genetic variability in Anacardiaceae, showing that individuals in this group have high levels of genetic variability [37,38,39].

The high estimates of heritability in the broad sense (>90%) and CVg/CVe ratios greater than 1 for most traits indicate a predominance of genetic variability over environmental influence under the conditions evaluated, suggesting high potential for response to selection [40]. Among the traits evaluated, fruit length and diameter, fresh fruit weight, and endocarp length and weight stood out as the descriptors with the greatest potential for use in umbuzeiro plant breeding programs, as they presented high heritability and strong genetic control. These variables correlate positively with seedling emergence and vigor, indicating that larger fruits and heavier endocarps tend to produce seeds with greater physiological vigor. Thus, morphological descriptors prove to be effective tools in identifying genetic variability, although they do not fully replace molecular approaches, which remain essential for more accurate genetic characterization [12].

Umbu fruits are commercially valuable, and their endocarps are essential for seedling production and genetic diversity. Each umbu seed is surrounded by a rigid pyrenium, which acts as a dispersal unit composed of fibrous-woody material with variations in shape and size [9,23], making morphological characterization an essential tool for the preliminary selection of promising genotypes [41]. Biometric analysis of *S. tuberosa* fruits revealed that the genotypes of the three populations studied (4, 9, 18, 27, 30, 33, and 34) presented notable traits, such as length, diameter, and fresh mass. These trees have the potential to increase fruit production, both for commercial markets and for fresh consumption. The biometric data of the fruits obtained in this study were similar to those reported in previous studies [11,37,41,42,43], with lengths ranging from 24.9 to 40.9 mm, diameters ranging from 23.3 to 38.6 mm, and fresh weights ranging from 9.2 to 30.2 g.

The variations observed among *Spondias tuberosa* populations may be associated not only with genetic differences, but also with the influence of local edaphoclimatic factors. The heterogeneity of soil types— Regossolo Distrófico in Algodão de Jandaíra and Solonetz Solodizado in Boa Vista and São José da Mata—combined with variation in annual precipitation (387.5–777.0 mm) may have contributed to the differentiated expression of morphological and physiological traits of the fruits. These environmental conditions possibly increase the phenotypic plasticity of the species, affecting fruit size and mass, and should be considered in the interpretation of the differences observed between genotypes, since they reflect the genotype × environment interaction.

Comparing the fruits of *S. tuberosa* from populations in Paraíba with those from other regions is extremely important for various scientific and practical aspects. Comparisons between populations help identify phenotypic differences and genetic variability, which are essential for the adaptability of the species and the development of improved cultivars, adding value to local products and benefiting the economy and sustainable production [24,37,41,44]. In general, the physical traits of fruits, such as their shape, circularity, and surface texture, are fundamental to their commercial viability, especially in the pulp industry [9]. In this sense, Santana et al. [9] and Lins Neto et al. [10] reported considerable genetic and morphological diversity in *S. tuberosa* populations in the semiarid region of northeastern Brazil. The authors emphasized that phenotypic data are fundamental to understanding the genetic diversity and ecological adaptation of the species, as well as assisting in the selection of breeding programs that increase productivity and resistance to abiotic stresses.

The dimensions of the endocarps varied among the three plant populations, both in length, diameter, and thickness, as well as in fresh mass, such that significant variability in endocarp size was observed among seeds from the same tree, which, according to Dutra et al. [43], is common in propagation by sexual reproduction. When marketing fruits, smaller endocarps are preferable; however, larger endocarps may contain larger seeds with more reserves for germination, making them more suitable for seedling production [45]. These observations highlight the role of endocarp size and mass and seed nutritional reserves associated with seedling emergence, emphasizing their ecological importance in plant development and survival strategies.

There was variation in the percentage of seedling emergence among the populations of *S. tuberosa* in Algodão de Jandaíra (11%), Boa Vista (25%), and São José da Mata (30%), which may indicate differences in the germination potential and ecological adaptation of seeds from these regions. When these percentages are compared with those of seeds from freshly harvested fruits in previous studies [9,46,47,48], although they are within the reported ranges, the averages of the diaspores of the São José da Mata population tend to approach the highest values. Within this population, genotype 9 stands out, with an emergence rate of 75%, indicating that its descendants can be used as rootstocks owing to their higher probability of germination. This genotype showed considerable values for fruit dimensions—length of 38.84 mm, diameter of 33.90 mm, and mass of 26.07 g—while the dimensions and mass of the endocarp were relatively modest, with a length of 21.46 mm, width of 13.81 mm, thickness of 11.07 mm, and mass of 1.37 g. These results reinforce the prominence of this genotype due to the balance between good fruit development and germination efficiency, pointing to it as promising material for use as rootstock in *Spondias tuberosa*.

The low germination rate of the diaspores of this species may be related to some type of dormancy or intrinsic factors of the tree’s genetics that affect seed vigor. Notably, in our study, no pre-germination method was used to evaluate natural germination, allowing for a more accurate analysis of germination behavior under conditions similar to those of the natural environment.

In our study, similarities were observed between the three populations of *S. tuberosa* with respect to landscape management areas, such as the presence of individuals in agricultural habitats, suggesting possible domestication. Although the domestication of species of the genus *Spondias* may reduce genetic variation in cultivated populations compared with wild populations, as observed in *Spondias purpurea* L. [38], many wild or early domesticated species, such as *S. tuberosa*, retain high genetic variability [10]. On the other hand, the domestication of species, supported by the traditional knowledge of local communities, promotes the selection of varieties with desirable traits, such as increased productivity and resistance. These communities are fundamental for conserving genetic diversity and maintaining practices that favor variability, which is essential for the adaptation of species to environmental changes and human needs.

The formation of 12 groups using the Tocher method and 6 using UPGMA demonstrates broad genetic divergence between genotypes, but the presence of individuals from different populations in the same groups indicates that variability occurs mainly within populations. This pattern reflects the gene flow promoted by allogamous reproduction and zoochoric dispersal of diaspores, which favor genetic mixing between individuals. Thus, the grouping structure confirms that the diversity of *Spondias tuberosa* is predominantly intrapopulational, a characteristic typical of perennial and allogamous species of the Caatinga. These results corroborate the studies by Silva et al. [49] and Zortéa et al. [39] on populations of *Spondias mombin* L., which reported high genetic diversity, which was greater within populations than between them. Notably, many *Spondias* species occur *circa situm*, a term that refers to conservation strategies where planted or remnant species are maintained in agricultural landscapes and can maintain levels of genetic diversity similar to those of wild populations [50].

Although the populations studied show high intrapopulation diversity, it is important to consider that habitat fragmentation and the reduction in natural dispersers in the Caatinga may restrict gene flow between populations. The loss of ecological connectivity caused by agricultural expansion and urbanization may intensify geographical isolation and inbreeding, affecting the maintenance of genetic variability [51]. However, the structure observed in the groupings suggests that genetic exchange between nearby populations still exists, possibly facilitated by zoochoric agents and traditional management by local communities. This scenario reinforces the need for measures to reconnect fragments, such as ecological corridors and mixed genetic reforestation, in order to mitigate isolation and promote continuous gene flow.

*S. tuberosa* trees have an aggregated distribution, indicating that a significant number of diaspores are deposited near the parent plant [11], possibly facilitated by zoochoric dispersal. However, the decline in natural disperser populations caused by the degradation of the Caatinga biome has resulted in a significant decline in *S. tuberosa* populations [52]. This trend is concerning because long-distance fruit dispersal is a factor that favors greater genetic variation within tree species populations [53].

The correlation analysis between fruit and endocarp traits revealed important relationships that will aid in the genetic improvement of the species, with significant implications for the management of *S. tuberosa* populations and fruit harvesting, since the selection of trees that produce fruits with greater fresh mass can optimize harvesting for the market and the cultivation of productive forests, improving the income of communities and contributing to the conservation of *S. tuberosa* in its natural habitat [11]. In addition, positive correlations between fruit and endocarp dimensions and seedling emergence indicate that larger and heavier diaspores tend to exhibit higher germination and vigor, providing valuable information for selection and conservation strategies. To enable agroindustrial and medicinal exploitation of *S. tuberosa*, it is necessary to intensify scientific efforts in propagation and management practices, shorten the time between germination and fruiting, and understand its problematic reproduction to protect its genetic diversity [22].

The unique groups identified by the Tocher and UPGMA methods (genotypes 9, 14, 17, and 19) represent genetically distinct materials and are therefore priorities for germplasm collection and the formation of ex situ conservation banks, ensuring the representativeness of genetic diversity. Thus, the observed variability not only supports the selection of genotypes with larger fruits and more vigorous seeds, but also guides practical genetic conservation strategies. This genetic diversity also reflects the high adaptive potential of *S. tuberosa* to semiarid conditions [9,21]. Populations with wide phenotypic variability tend to have greater ecological plasticity, a fundamental characteristic for tolerating water and temperature fluctuations, which are common in the Caatinga. This diversity acts as an evolutionary reserve, enabling the species to respond differently to environmental pressures, maintaining its ecological and reproductive viability in scenarios of climate change [12]. Thus, the results of this study show that maintaining genetic variability is essential to ensure the adaptive resilience of *S. tuberosa*, sustaining both the ecosystem and the local bioeconomies associated with its use.

Considering the results obtained and the conservation challenges faced by the species, it is recommended that integrated in situ and ex situ conservation strategies be adopted, combining technical and community actions. Among the proposed measures are: (i) targeted collection of germplasm, prioritizing unique and more divergent genotypes; (ii) creation of regional seed and seedling banks, ensuring genetic representativeness; (iii) strengthening of *circa situm* conservation programs in agricultural and community use areas; (iv) rotation of collections and preservation of parent trees to promote natural regeneration; and (v) implementation of educational and participatory actions that value the cultural and economic role of the umbuzeiro tree. These practices are fundamental to ensuring the sustainability of the species, aligning genetic conservation, ecological restoration, and socio-environmental development in the Brazilian semiarid region.

## 5. Conclusions

The genetic diversity of *Spondias tuberosa* populations enables the targeted collection of diaspores for conservation and genetic improvement, which is essential for maintaining their evolutionary potential and facilitating the collection of germplasm in ex situ conservation.

Fruit length is the phenotypic characteristic that contributes the most to explaining the dissimilarity between the genotypes evaluated.

The divergence observed between and within populations indicates that to adequately represent diversity, it is necessary to sample individuals from different groups.

Although fragmentation and habitat loss in the Caatinga may increase inbreeding and phenotypic similarity in *S. tuberosa* populations, the observed high genetic variability indicates that these populations are likely resilient to genetic erosion caused by habitat fragmentation.

## Figures and Tables

**Table 1:** Environmental characteristics of *Spondias tuberosa* Arruda (Anacardiaceae) populations in Caatinga areas in Paraíba, Brazil.

Population	Number of Genotypes	Altitude (masl.)	Average Temperature (min.–max.) (°C)	Average Annual Precipitation (mm)	Soil Type
Algodão de Jandaíra	8	470	23.3 (19.3–29.5)	387.5	Regossolo Distrófico
Boa Vista	17	493	23.4 (19.3–29.8)	418.8	Solonetz Solodizado
São José da Mata	13	551	23.5 (20.2–28.8)	777.0	Solonetz Solodizado

**Table 2:** Analysis of variance of the physical and physiological traits of *Spondias tuberosa* Arruda (Anacardiaceae) diaspores from three natural populations in Caatinga areas in Paraíba, Brazil.

**Variation Factor**	**Mean Squares**				
	**FL (mm)**	**FD (mm)**	**FFM (g)**	**EL (mm)**	**EW (mm)**
Genotypes	37.29 **	28.71 **	74.84 **	9.47 **	5.53 **
Residue	0.55	0.65	1.81	0.41	0.27
h^2^ (%)	98.51	97.70	97.57	95.60	94.96
CVg/CVe	4.07	3.26	3.17	2.33	2.17
CV (%)	2.17	2.60	6.52	3.27	3.82
**Variation Factor**	**Mean Squares**				
	**ET (mm)**	**EM (g)**	**SE (%)**	**ESI**	**MET (days)**
Genotypes	3.24 **	0.16 **	1061.12 **	0.07 **	720.45 **
Residue	0.24	0.01	99.40	0.01	265.81
h^2^ (%)	92.32	96.0	90.63	91.79	63.10
CVg/CVe	1.73	2.44	1.55	1.67	0.65
CV (%)	4.60	6.87	42.52	48.75	36.41

(**) Significant effect at 1% probability according to the *F* test. Legend: Heritability (h^2^); ratio between genetic and environmental coefficients of variation (CVg/CVe); coefficient of variation (CV); fruit length (FL); fruit diameter (FD); fresh fruit mass (FFM); endocarp length (EL); endocarp width (EW); endocarp thickness (ET); endocarp mass (EM); seedling emergence (SE); emergence speed index (ESI); mean emergence time (MET).

**Table 3:** Clustering of 38 genotypes of *Spondias tuberosa* Arruda (Anacardiaceae) via the Tocher optimization method, which is based on the standardized mean Euclidean distance, estimated from ten quantitative characters of the diaspores.

Groups	Genotypes ^1^
I	1–5–11–13–20–21–22–27–28–29–31–35–36–37
II	2–3–6–8–10–12–32
III	24–25–26
IV	4–7–18–30
V	15–16–38
VI	14
VII	23
VIII	17
IX	19
X	33
XI	9
XII	34

^1^ Identification of genotypes and their respective populations: 1 to 13: São José da Mata, 14 to 21: Algodão de Jandaíra, 22 to 38: Boa Vista.

**Table 4:** Pearson correlation (*r*) between the phenotypic and physiological traits of *Spondias tuberosa* Arruda (Anacardiaceae) diaspores.

Variables	FD	FFM	EL	EW	ET	EM	SE	ESI	MET
FL	0.911 **	0.902 **	0.766 **	0.252 ^ns^	0.161 ^ns^	0.408 *	0.414 **	0.397 *	0.059 ^ns^
FD		0.951 **	0.720 **	0.492 **	0.368 *	0.514 **	0.279 ^ns^	0.255 ^ns^	0.110 ^ns^
FFM			0.720 **	0.432 **	0.329 *	0.468 **	0.267 ^ns^	0.254 ^ns^	0.107 ^ns^
EL				0.585 **	0.608 **	0.680 **	0.412 **	0.419 **	−0.071 ^ns^
EW					0.882 **	0.697 **	0.176 ^ns^	0.134 ^ns^	0.115 ^ns^
ET						0.692 **	0.116 ^ns^	0.143 ^ns^	−0.090 ^ns^
EM							0.329 **	0.326 *	−0.056 ^ns^
SE								0.920 **	−0.030 ^ns^
ESI									−0.332 *

* (*p* < 0.05), ** (*p* < 0.01), (^ns^) Not significant. FL: fruit length; FD: fruit diameter; FFM: fresh fruit mass; EL: endocarp length; EW: endocarp width; ET: endocarp thickness; EM: endocarp mass; SE: seedling emergence; ESI: emergence speed index; MET: mean emergence time.

## Data Availability

The original contributions presented in the study are included in the article. The data presented in this study are available on request from the corresponding author.

## Supplementary Materials

The following supporting information can be downloaded at https://www.mdpi.com/article/10.3390/biology14121641/s1: Table S1: Means of physical and physiological traits of the diaspores of 38 genotypes of *Spondias tuberosa* Arruda (Anacardiaceae) from three natural populations in Caatinga areas in Paraíba, Brazil.

## Abbreviations

The following abbreviations are used in this manuscript:

AFLP	Amplified Fragment Length Polymorphism
ANOVA	Analysis of variance
CV	Coefficient of variation
CVe	Environmental coefficient of variation
CVg	Genetic coefficient of variation
EL	Endocarp length
EM	Endocarp mass
ESI	Emergence speed index
ET	Endocarp thickness
EW	Endocarp width
FD	Fruit diameter
FFM	Fresh fruit mass
FL	Fruit length
GPS	Global positioning system
ISSR	Inter-simple Sequence Repeat
MET	Mean emergence time
RAPD	Random Amplified Polymorphic DNA
SDTFs	Seasonally dry tropical forests
SNP	Single-Nucleotide Polymorphisms
SE	Seedling emergence
UPGMA	Unweighted Pair Group Method using Arithmetic Averages

## Appendix A

### Appendix A.1

**Table A1:** Averages of phenotypic traits of *Spondias tuberosa* Arruda (Anacardiaceae) diaspores for each group according to Tocher’s optimization method.

**Group**	**Phenotypic Traits ^1^**				
	**FL (mm)**	**FD (mm)**	**FFM (g)**	**EL (mm)**	**EW (mm)**
I	33.54 (30.06–36.63)	30.87 (27.75–34.10)	20.41 (16.65–25.58)	18.92 (17.10–20.47)	13.55 (12.13–14.77)
II	34.94 (32.47–36.97)	30.88 (28.46–32.67)	20.12 (16.09–23.72)	20.28 (19.24–21.78)	13.07 (12.28–13.87)
III	33.87 (32.57–35.01)	32.57 (30.67–34.36)	21.28 (18.58–24.94)	20.18 (19.60–21.29)	15.57 (14.85–16.41)
IV	37.62 (36.13–38.96)	34.45 (33.78–34.95)	27.04 (24.08–29.03)	21.18 (20.88–21.62)	14.14 (13.38–14.92)
V	29.84 (28.95–30.35)	28.08 (27.11–29.65)	15.28 (13.38–16.83)	17.59 (16.79–18.05)	13.16 (12.40–14.03)
VI	25.15	23.60	9.29	16.73	12.67
VII	30.86	26.91	14.70	18.02	12.27
VIII	33.11	27.86	15.22	20.62	14.28
IX	34.66	33.52	23.85	22.00	17.45
X	37.88	35.16	23.45	21.70	15.56
XI	38.84	33.90	26.07	21.47	13.81
XII	40.17	34.64	27.31	22.76	14.53
**Group**	**ET (mm)**	**EM (g)**	**SE (%)**	**ESI**	**MET (days)**
I	10.40 (8.61–11.07)	1.09 (0.90–1.33)	16 (1–32)	0.10 (0–0.25)	46 (19–48)
II	10.63 (9.97–11.60)	1.11 (0.82–1.47)	26 (12–36)	0.20 (0.06–0.27)	39 (23–53)
III	12.03 (11.30–12.94)	1.34 (1.29–1.45)	37 (27–44)	0.19 (0.17–0.22)	57 (48–71)
IV	11.31 (10.40–11.89)	1.34 (1.11–1.50)	22 (5–36)	0.16 (0.02–0.33)	47 (31–73)
V	10.90 (10.33–11.83)	1.01 (0.95–1.05)	12 (3–30)	0.09 (0.02–0.24)	43 (35–53)
VI	10.56	1.19	5	0.04	33
VII	9.28	0.80	25	0.11	64
VIII	11.09	1.27	55	0.43	36
IX	13.23	1.60	4	0.11	40
X	11.34	1.48	46	0.34	38
XI	11.07	1.38	75	0.71	29
XII	11.16	1.34	39	0.21	57

^1^ FL: fruit length; FD: fruit diameter; FFM: fresh fruit mass; EL: endocarp length; EW: endocarp width; ET: endocarp thickness; EM: endocarp mass; SE: seedling emergence; ESI: emergence speed index; MET: mean emergence time. The data are presented as averages (minimum–maximum).

### Appendix A.2

**Table A2:** Averages of phenotypic traits of *Spondias tuberosa* Arruda (Anacardiaceae) diaspores for each group according to UPGMA method.

**Group ^1^**	**Phenotypic Traits**				
	**FL (mm)**	**FD (mm)**	**FFM (g)**	**EL (mm)**	**EW (mm)**
I	34.22 (28.95–40.17)	31.28 (26.91–35.16)	20.89 (13.38–29.03)	19.71 (16.79–22.76)	13.76 (12.17–16.41)
II	25.15	23.60	9.29	16.73	12.67
III	33.11	27.86	15.22	20.62	14.28
IV	35.48	32.68	20.41	17.10	12.13
V	34.66	33.52	23.85	22.00	17.45
VI	38.84	33.90	26.07	21.47	13.81
**Group**	**ET (mm)**	**EM (g)**	**SE (%)**	**ESI**	**MET (days)**
I	10.83 (9.28–12.94)	1.15 (0.80–1.50)	22 (1–46)	0.15 (0–0.34)	46 (19–73)
II	10.56	1.19	5	0.04	33
III	11.09	1.27	55	0.43	36
IV	8.61	1.25	19	0.09	63
V	13.23	1.60	4	0.11	40
VI	11.07	1.38	75	0.71	29

^1^ Groups II, III, IV, V, and VI were formed solely by genotypes 14, 17, 22, 19, and 9, respectively, and group I by the remaining genotypes. FL: fruit length; FD: fruit diameter; FFM: fresh fruit mass; EL: endocarp length; EW: endocarp width; ET: endocarp thickness; EM: endocarp mass; SE: seedling emergence; ESI: emergence speed index; MET: mean emergence time. The data are presented as averages (minimum–maximum).
