# Supplementary material for: Phenotypic Expression of Diaspores Indicates Genetic Diversity in Natural Populations of Spondias tuberosa (Anacardiaceae)
Source: Biology (Basel). 2025 Nov 21;14(12):1641. doi: 10.3390/biology14121641 (PMC12729702; doi:10.3390/biology14121641)
Supplement: Supplementary file 1 [file biology-14-01641-s001.zip › biology-3941628-supplementary.pdf]

Supplementary Material

Table S1. Means of quantitative traits related to the physical and physiological traits of the diaspores of 38 genotypes of *Spondias tuberosa* Arruda (Anacardiaceae) from three natural populations in Caatinga areas in Paraíba, Brazil.

Genotype	Fruit length (mm)	Fruit diameter (mm)	Fresh fruit mass (g)	Endocarp length (mm)	Endocarp width (mm)	Endocarp thickness (mm)	Endocarp mass (g)	Seedling emergence (%)	ESI	MET
**1**	35.18 d	31.37 b	20.20 d	19.53 d	13.42 e	10.55 d	1.06 e	22 c	0.22 c	27.21 b
**2**	34.17 e	30.80 c	19.60 e	20.77 c	13.66 e	11.31 c	1.23 d	27 c	0.23 c	32.75 b
**3**	32.47 f	28.45 e	16.09 f	19.25 d	12.28 f	9.97 d	0.97 f	18 c	0.12 c	44.34 b
**4**	38.95 b	34.94 a	29.02 a	21.00 c	13.37 e	10.40 d	1.11 e	29 c	0.23 c	36.15 b
**5**	33.19 f	29.93 d	19.01 e	18.58 e	13.71 e	10.80 d	1.07 e	20 c	0.13 d	43.72 b
**6**	34.02 e	30.00 d	19.15 e	19.24 d	12.37 f	10.07 d	0.91 f	31 c	0.25 c	35.20 b
**7**	36.12 c	33.77 a	24.08 c	20.88 c	14.34 d	11.90 b	1.36 c	36 c	0.32 b	30.81 b
**8**	36.91 c	32.34 b	23.71 c	21.30 b	13.70 e	11.12 c	1.23 d	36 c	0.24 c	42.94 b
**9**	38.84 b	33.90 a	26.07 b	21.46 b	13.81 e	11.07 c	1.37 c	75 a	0.71 a	28.50 b
**10**	35.88 d	31.62 b	21.97 d	21.78 b	13.86 e	11.60 b	1.46 b	30 c	0.18 c	45.00 b
**11**	35.42 d	31.50 b	20.83 d	18.92 e	12.17 f	9.57 e	1.07 e	15 d	0.11 d	39.92 b
**12**	34.17 e	30.30 c	18.57 e	20.13 c	12.57 f	10.25 d	1.10 e	29 c	0.26 c	23.17 b
**13**	30.05 g	27.75 f	16.09 f	18.56 e	13.87 e	11.07 c	1.11 e	17 c	0.11 d	42.51 b
**14**	25.14 i	23.60 g	9.28 h	16.73 f	12.67 f	10.56 d	1.85 d	5 d	0.03 d	32.75 b
**15**	30.23 g	27.10 f	13.38 g	17.94 e	12.40 f	10.53 d	1.05 e	4 d	0.02 d	53.00 a
**16**	30.34 g	29.64 d	16.82 f	18.04 e	14.03 e	11.82 b	1.04 e	3 d	0.01 d	35.25 b
**17**	33.10 f	27.86 f	15.22 g	20.61 c	14.28 d	11.10 c	1.27 d	55 b	0.43 b	35.58 b
**18**	38.45 b	34.78 a	28.10 a	21.22 b	13.90 e	11.24 c	1.38 c	5 d	0.02 d	46.25 b
**19**	34.65 e	33.51 a	23.85 c	22.00 b	17.44 a	13.23 a	1.59 a	4 d	0.11 d	39.75 b
**20**	33.30 f	29.95 d	18.26 e	19.80 d	12.97 f	10.38 d	1.04 e	2 d	0.009 d	28.75 b
**21**	33.42 f	32.00 b	20.85 d	20.47 c	14.07 e	10.97 c	1.14 e	6 d	0.03 d	47.38 b
**22**	35.48 d	32.67 b	20.40 d	17.10 f	12.12 f	8.60 f	1.25 d	19 c	0.09 d	62.70 a
**23**	30.86 g	26.90 f	14.70 g	18.02 e	12.28 f	9.27 e	0.80 g	25 c	0.10 d	63.69 a
**24**	34.02 e	32.70 b	20.32 d	19.64 d	15.45 c	11.85 b	1.29 c	40 b	0.22 c	53.99 a
**25**	35.01 d	34.36 a	24.94 c	21.28 b	16.41 b	12.94 a	1.44 b	27 c	0.17 c	47.80 b
**26**	32.78 f	30.66 c	18.58 e	19.60 d	14.84 d	11.30 c	1.29 c	44 b	0.16 c	70.54 a

Table S1. Continuation…

Genotype	Fruit length (mm)	Fruit diameter (mm)	Fresh fruit mass (g)	Endocarp length (mm)	Endocarp width (mm)	Endocarp thickness (mm)	Endocarp mass (g)	Seedling emergence (%)	ESI	MET
**27**	36.62 c	34.10 a	25.57 b	19.14 d	13.70 e	10.61 d	1.00 e	28 c	0.11 d	67.73 a
**28**	32.86 f	30.73 c	20.97 d	19.29 d	14.08 e	10.62 d	0.96 f	8 d	0.04 d	59.70 a
**29**	32.78 f	30.70 c	20.41 d	18.78 e	14.36 d	10.74 d	0.90 f	15 d	0.06 d	58.48 a
**30**	36.94 c	34.30 a	26.94 b	21.61 b	14.92 d	11.72 b	1.49 b	19 c	0.07 d	73.40 a
**31**	32.29 f	30.83 c	20.43 d	18.56 e	14.10 e	10.98 c	1.20 d	1 d	0.003 d	18.50 b
**32**	36.97 c	32.67 b	21.71 d	19.48 d	13.02 f	10.07 d	0.81 g	12 d	0.05 d	53.02 a
**33**	37.87 b	35.16 a	23.45 c	21.70 b	15.55 c	11.34 c	1.47 b	46 b	0.34 b	38.42 b
**34**	40.17 a	34.63 a	27.31 b	22.76 a	14.52 d	11.15 c	1.33 c	39 b	0.20 c	56.52 a
**35**	32.37 f	29.69 d	19.47 e	19.55 d	14.76 d	10.92 c	1.32 c	30 c	0.15 c	55.80 a
**36**	31.90 f	28.80 e	18.00 e	17.39 f	12.75 f	9.68 e	0.91 f	7 d	0.04 d	52.67 a
**37**	34.59 e	32.11 b	24.61 c	19.18 d	13.53 e	10.15 d	1.17 d	32 c	0.24 c	35.66 b
**38**	28.95 h	27.48 f	15.64 f	16.80 f	13.05 f	10.32 d	0.94 f	30 c	0.23 c	41.65 b

[1] Plant populations: 1–13 (São José da Mata); 14–21 (Algodão de Jandaíra); 22–38 (Boa Vista). Means followed by the same letter in the column do not differ from each other according to the Scott–Knott test at 5% probability. ESI: emergence speed index; MET: mean emergence time.
